# Quality indicators for community care for older people: A systematic review

**DOI:** 10.1371/journal.pone.0190298

**Published:** 2018-01-09

**Authors:** Karlijn J. Joling, Liza van Eenoo, Davide L. Vetrano, Veerle R. Smaardijk, Anja Declercq, Graziano Onder, Hein P. J. van Hout, Henriëtte G. van der Roest

**Affiliations:** 1 Department of General Practice and Elderly Care Medicine, Amsterdam Public Health Research Institute, VU University Medical Center, Amsterdam, The Netherlands; 2 LUCAS, KU Leuven, University of Leuven, Leuven, Belgium; 3 Department of Geriatrics, Centro Medicina dell’Invecchiamento, Universita`Cattolica Sacro Cuore, Rome, Italy; 4 Aging Research Center, Karolinska Institutet, Stockholm, Sweden; 5 Stockholm University, Stockholm, Sweden; Universita degli Studi di Ferrara, ITALY

## Abstract

**Background:**

Health care systems that succeed in preventing long term care and hospital admissions of frail older people may substantially save on their public spending. The key might be found in high-quality care in the community. Quality Indicators (QIs) of a sufficient methodological level are a prerequisite to monitor, compare, and improve care quality. This systematic review identified existing QIs for community care for older people and assessed their methodological quality.

**Methods:**

Relevant studies were identified by searches in electronic reference databases and selected by two reviewers independently. Eligible publications described the development or application of QIs to assess the quality of community care for older people. Information about the QIs, the study sample, and specific setting was extracted. The methodological quality of the QI sets was assessed with the Appraisal of Indicators through Research and Evaluation (AIRE) instrument. A score of 50% or higher on a domain was considered to indicate high methodological quality.

**Results:**

Searches resulted in 25 included articles, describing 17 QI sets with 567 QIs. Most indicators referred to care processes (80%) and measured clinical issues (63%), mainly about follow-up, monitoring, examinations and treatment. About two-third of the QIs focussed on specific disease groups. The methodological quality of the indicator sets varied considerably. The highest overall level was achieved on the domain ‘Additional evidence, formulation and usage’ (51%), followed by ‘Scientific evidence’ (39%) and ‘Stakeholder involvement’ (28%).

**Conclusion:**

A substantial number of QIs is available to assess the quality of community care for older people. However, generic QIs, measuring care outcomes and non-clinical aspects are relatively scarce and most QI sets do not meet standards of high methodological quality. This study can support policy makers and clinicians to navigate through a large number of QIs and select QIs for their purposes.

PROSPERO Registration: 2014:CRD42014007199

## Introduction

In the world’s aging population, a growing number of older people will lead to a rapid increase in the demand for health care services. At the same time, a shortage of professional and informal caregivers is projected [1, 2]. Policy makers need to anticipate on these trends and prepare health care systems to function as efficiently as possible in order to serve all future older citizens with appropriate and affordable care.

A majority of older people prefers to remain living at home for as long as possible and receive care at home when needed [3, 4]. In many countries, current policies aim to follow up on this preference and strongly promote the use of community-based services, which is also expected to help keeping health care sustainable. Almost 40% of public spending on health care concerns persons over 65 years of age, with long term care and hospital admissions being the most important cost drivers [5, 6]. Health care systems that succeed to provide effective community-based care and services are likely to optimise their public spending substantially [7].

As a result, community care services are becoming more important for older people to rely on. To monitor and stimulate high-quality community care, valid indicators are a prerequisite for being able to identify where, when and under which conditions quality deficiencies exist. Quality Indicators (QIs) are measurable elements of practice performance for which there is evidence or consensus that they can be used for assessing and changing the provided quality of care [8]. They can provide quantified indications for various stakeholders. Clinicians can use them for bench learning, and to set priorities for improvement and education. QIs can also provide transparency about the quality of care delivery and the performance of care professionals for patients (or their representatives). Health care insurers, Ministries of Health, and Health Care Inspectorates can use QIs for monitoring, supervision and policy making.

According to Donebedian’s widely used model for assessing health care quality, QIs can be related to process, outcomes and structure of care. Process indicators denote what is actually done while giving and receiving care, for example developing a care plan, or conducting an annual medication review. Structure indicators involve the attributes of the care setting, such as materials and resources [9]. Outcome indicators describe the effect of care on patients’ health status, such as a reduction in pain since intake, or improved quality of life. Following this model, patient satisfaction can be defined as a patient-reported outcome measure, while the structures and processes of care can be measured by patient-reported experiences [10]. There is debate about the most useful types of indicators to assess the quality of care. Process indicators are direct measures of quality, are considered to be more sensitive to differences in the quality of care, and can be more straightforward to interpret without extensive risk adjustment. On the other hand, outcome indicators reflect the interplay of a wide variety of factors and are of greater intrinsic interest as they assess the effect of health care services on desired outcomes [11, 12].

Regardless of whether structural, process or outcome indicators are chosen, it is important that QIs adhere to certain quality requirements to produce an accurate measure of quality. Criteria of the National Quality Forum are widely recognized as important for evaluating quality indicators and include ‘importance’, ‘scientific acceptability of measure properties’, ‘usability’, and ‘feasibility’ [13]. ‘Importance’ covers the extent to which the focus of the QI is evidence-based and important to making significant improvements in healthcare quality where there is variation in or overall less-than-optimal performance [13]. Where possible, this should be based directly upon rigorous scientific evidence. When such evidence is absent, consensus techniques and guideline driven approaches can be used [14]. The criterion ‘Scientific acceptability’ requires that the QI is well defined and precisely specified, and addresses whether it produces consistent (reliable) and credible (valid) results about the quality of care when implemented. Additional requirements, such as specification of a risk adjustment strategy to account for case-mix differences, are also included within this criterion. Feasibility depends on the extent to which the required data are readily available or could be obtained without excessive burden and demonstrates whether the data collection strategy can be implemented. Lastly, ‘usability’ represents the extent to which potential stakeholder groups are using or could use the results for both accountability and quality improvement [13].

Although these criteria clearly indicate the key quality requirements for indicators, in a real world, the quality of QIs varies considerably, which hinders meaningful reflection and comparison of quality of care. An overview of QIs that are available to measure the quality of care for older persons in community care settings (e.g. primary care and home care services) and the extent to which these QIs meet quality requirements is currently lacking. Knowing which QIs are available, and having insight in their characteristics and methodological quality can support relevant stakeholder groups in selecting the right indicators for their quality purposes, and prevent the development of new indicators for quality domains that are already covered sufficiently. Such an overview will also identify shortcomings in QIs that are currently being applied, along with giving guidance for further development or improvement. Therefore, the objectives of this systematic review are to provide a comprehensive overview of existing QIs developed or applied to assess the quality of community care provided to older people, to differentiate between types of indicators, and to evaluate the methodological quality of the identified QI sets.

## Materials and methods

The protocol for this systematic review has been published on PROSPERO (2014:CRD42014007199) and is available at: http://www.crd.york.ac.uk/PROSPERO/display_record.asp?ID=CRD42014007199. The PRISMA guidelines for reporting systematic reviews were used in undertaking the review.

### Search strategy

A search strategy was developed to identify publications concerning the development, testing or implementation of indicators of the quality of community care for older people. Searches were conducted in consultation with a librarian in electronic reference databases (Medline, PsycINFO, EMBASE, CINAHL and Cochrane) on September 25, 2013, with no restriction to language or publication year. We combined key words and medical-subject headings for home care, quality indicators and older-aged people. S1 Appendix presents the search strategy in Medline. Comparable searches were performed in the other databases and are available on request. The searches in the databases were updated up to November 21^st^, 2016 to examine if new QI sets meeting the inclusion criteria had appeared since our initial search.

### Study selection

Publications were included if:

1. They described the development and/or characteristics of QIs specifically developed for older people or applied in an older aged sample (i.e. 65 years or older). Disease specific QI sets should have a specific connection to older people (e.g. focus on core geriatrics topics, such as falls or dementia). If there was no clear connection, the QI set should have an explicit goal of measuring the quality of care in older people with the condition in question.
2. The QIs were developed or applied to assess the quality of care in the community (e.g. home care, primary care, community care and ambulatory care).
3. Numerators and denominators of the QIs were defined or could be deduced from the descriptions of the QIs.

Editorials, letters to the editor, comments, narrative case-reports and articles written in a language other than English, Dutch, German or Italian were excluded. When a set of QIs was updated, we selected the publication describing the updated QIs. The identified references were entered in a bibliographical database and duplicates were removed. First, the title and abstracts of these references were assessed for relevance by two reviewers independently (KJ and LvE, DV or VS). Next, the full text of the selected references was obtained and reviewed by two reviewers independently (KJ and LvE, DV or VS). Any disagreements between reviewers were resolved by consensus. If no consensus could be reached a third reviewer (HvdR) was consulted. The reference lists of the obtained full-text publications were checked to identify any relevant publications that had not been identified in the searches. In addition, we solicited several researchers evaluating the quality of community care for older people in Europe (www.ibenc.eu/) to identify additional unpublished or grey literature.

### Data extraction

A data extraction form was used to extract the following information about the QIs: the general description of the QI, the numerator and denominator, and if applicable its performance standard and exclusion criterion. Furthermore, the QIs were classified as either a structure, process, or outcome measure and were categorized into the domain(s) they covered. To give more insight into the areas that were addressed by the QIs, we searched for an existing framework or domains of community care for older people that could be applied to categorize the QIs. As far as we are aware, such a classification or framework does not yet exist. Therefore, based on categorizations and useful (sub)headings used in the identified QI sets we included, the first author drafted a domain classification. The other research team members, with (clinical) backgrounds in geriatrics, psychology, sociology and epidemiology, commented on the draft and this resulted in a classification of the following nine domains:

1. *Clinical issues*, e.g. falls and mobility disorders, pain, ulcers, clinical conditions, nutrition, weight loss, dehydration, feeding tube, medications, tobacco and alcohol use, injuries, hearing and vision loss, clinical examinations, infections, mortality. Given the wide variety of clinical aspects covered in this domain, these QIs were further classified into the subcategories ‘screening and prevention’, ‘follow-up / monitoring and examinations’, clinical events and targets’ and ‘treatment (medication and non-pharmacological treatments)’.
2. *Cognition/ Mental health*, e.g. cognitive loss/ dementia, delirium, communication, mood, behavior, abuse.
3. *Structure of care*, e.g. budget resources, staff training, facilities and equipment.
4. *Continuity and coordination of care*, e.g. communication between care professionals, development of an individualized care plan.
5. *End of life care*, e.g. advanced care planning.
6. *Psycho-social aspects*, e.g. social activities, informal caregiving, loneliness.
7. *Service utilization*, e.g. hospitalizations, use of emergency services.
8. *Functional performance*, e.g. (Instrumental) Activities of Daily Living, home environment optimization, physical activities promotion, physical restraints.
9. *Patient perceptions and interaction*, e.g. satisfaction with care services, patient preferences.

### Methodological assessment

The methodological characteristics of the QI sets were assessed with the Appraisal of Indicators through Research and Evaluation (AIRE) Instrument [15]. The AIRE is a valid and reliable instrument specifically designed to appraise the quality of QIs [16]. It was derived from the Appraisal of Guidelines Through Research and Evaluation (AGREE) instrument [17], a widely used standard for assessing the methodological quality of practice guidelines. The AIRE has been used previously in several systematic reviews on QIs [18–22], and in studies developing QI sets [23, 24] for other patient groups. It includes 20 items that address four quality domains of a QI. Each item involves a statement about the quality of the QIs and is scored on a 4-point scale (1 ‘strongly disagree or no information provided’ to 4 ‘strongly agree’). The three domains reflecting the methodological quality were used to address the research objectives: ‘Stakeholder involvement’, ‘Scientific evidence’ and ‘Additional evidence formulation and usage’. Items of these domains were scored by two reviewers independently (KJ and VS) and summed per domain. Next, a standardized domain score was calculated according to the instrument’s guidelines with the following formula: (total score—minimum possible score) / (maximum score—minimum possible score) x 100%. A higher standardized score indicates a higher methodological level of quality (range 0–100%). QI sets were considered to have a high methodological quality on a domain if they scored 50% or higher, which correlates with an overall “agree” or “strongly agree” [21]. Domain scores are independent and should not be combined into a single quality score [15]. When more than one included article used (part of) the QI set, we incorporated all the information from these articles in the judgement. References were checked to be able to include information about for example the development process of QI sets. When a QI set was updated, we also examined the information from publication(s) which described the development of the original set for the scoring of the methodological quality.

## Results

### Selection of articles

**Fig 1:**
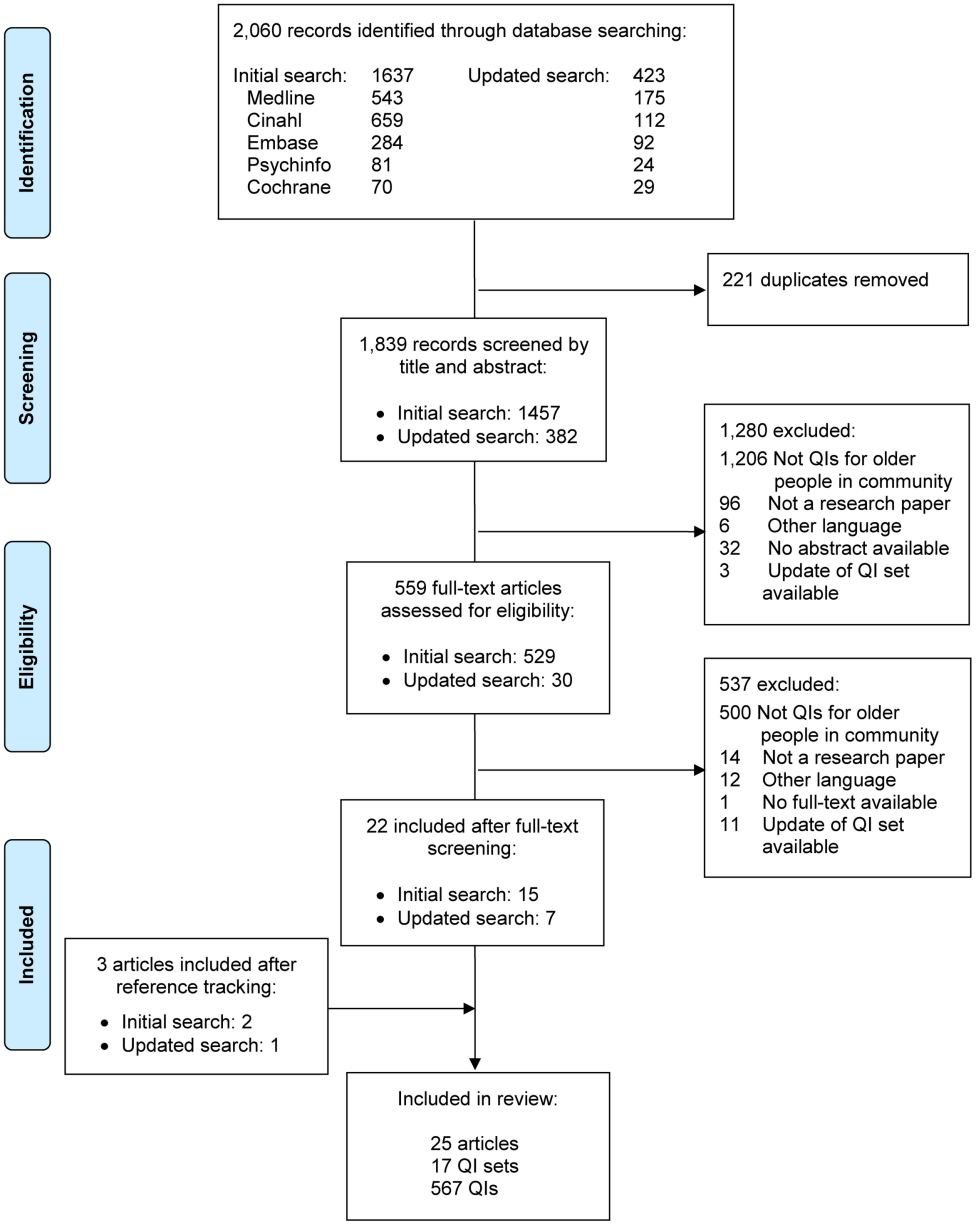
PRISMA flow diagram of the selection process.

A total of 1,839 unique publications were identified from the databases. Fig 1 presents a flow chart of the selection process and reasons for exclusion. After full-text screening, 22 articles met the selection criteria. Reference tracking of the publications identified three additional eligible articles. As a result, 25 articles were included in this review. These articles described a total of 17 QI sets, covering 567 unique QIs (S2 Appendix).

### Characteristics of the quality indicator sets

Table 1 presents a general overview of the included studies and the QI sets. Almost half of the studies originated from the USA (n = 12) [25–36], followed by the United Kingdom (n = 4) [37–40]. Other studies were from Canada [41], Taiwan [42], Sweden [43], The Netherlands [44–46], and two studies were performed in several European countries as part of an EU project [47, 48]. Six QI sets were developed or used in primary care settings [31, 37, 39, 45, 46, 49] and seven in home care settings [29, 38, 40, 42, 43, 47, 48]. Furthermore, one set was applied in a combination of primary care clinics and community agencies [34], two studies did not further specify community care [36, 41] and one set assessed the quality of ‘outpatient care’ for older people [28].

**Table 1:** Characteristics of the included quality indicator sets, presented by type of community care setting.

Author, year, (name QI set, if applicable)	Country	Aim QI set	Number of QIs	Setting	Study population (sample size)
*Home care settings*					
**Venables, 2006** [40]	United Kingdom	Performance of home care services for people with dementia	Total: 52 Outcome: 0 Process: 0 Structure: 52	Home care services	Older people with dementia and/or their informal carers receiving home care services (n = 113 services, with 229 clients)
**Kogan, 2010** [29]	USA	Nurse-physician communication	Total: 3 Outcome: 0 Process: 3 Structure: 0	Integrated home health care	Frail community-dwelling older adults with long-term care needs (sample size differed per QI and measurement from n = 30 to n = 394)
**Foebel, 2015** [48] (InterRai-Home Care QIs, 2^nd^ generation) [50]	Europe^a^	Home care services quality	Total: 23 Outcome: 22 Process: 1 Structure: 0	Home care organizations	Persons aged 65 and older receiving community care services (e.g. home care) for at least two weeks (n = 1,354)
**Jones, 2007** [38]	United Kingdom	Home care services quality (service user experiences)	Total: 2^b^ Outcome: 0 Process: 3 Structure: 0	Home care services	Home care service users aged 65 and older (n = 21,350)
**Chang, 2015** [42]	Taiwan	Quality of care for disabled older patients residing at home (vs. those residing in institutions)	Total: 5 Outcome: 5 Process: 0 Structure: 0	Home care (nursing care and doctors’ visits)	Disabled older patients aged 65 years and older who resided at home or in institutions and had submitted a first claim for coverage of National Health Insurance for home care over a 2-year period. Excluded: patients who received home care or institutional care within one year (365 days) before enrolment, died within three days of enrolment or transferred between home care and institutional care within one year of enrolment, or which records were without complete data (n = 27,894 in both the home care group and the institutional care group)
**Kajonius, 2016** [43]	Sweden	Structure quality and patient satisfaction with home care services	Total: 6 ^c^ Outcome: 0 Process: 4 Structure: 2	Home-based services	Older persons aged 65 years and older using home-based and nursing home care services (n = 61,600 in home care; n = 33,400 in nursing homes)
**Beerens, 2014** [47]	Europe ^d^	Quality of home care for people with dementia	Total: 8 ^e^ Outcome: 6 Process: 2 Structure: 0	Home care	People with dementia aged 65 years and older with an MMSE ≤24, who received home care and were at risk of admission to a long-term care facility within 6 months (n = 1,223 people living at home; n = 791 in institutional care)
*Primary care settings*					
**Fahey, 2003** [37]	United Kingdom	Three components of poor clinical care for elderly people: 1) insufficient use of beneficial drugs; 2) poor monitoring of chronic disease; 3) overuse of inappropriate or unnecessary drugs	Total: 15 Outcome: 2 Process: 13 Structure: 0	Primary care delivered by GPs	Elderly people aged 65 and older registered with general practices (people living at home were compared with people living in nursing homes). Excluded: Patients with terminal illness. (n = 526 people living at home; n = 172 in nursing homes)
**Lund, 2013** [31]	USA	Medication prescribing quality	Total: 4 Outcome: 3 Process: 1 Structure: 0	Primary care services	Older veterans aged 65 years and older, receiving Veterans Affairs primary care services (n = 1,549,824)
**Perry, 2010** [45]	Netherlands	Primary dementia care quality (diagnosis and management)	Total: 23 Outcome: 1 Process: 18 Structure: 4	Primary care (general practices)	Frail elderly people, suspected of suffering from cognitive problems (n = 63)
**Shah, 2011** [39]	United Kingdom	Chronic disease management (coronary heart disease,stroke, atrial fibrillation, and diabetes)	Total: 16 Outcome: 0 Process: 16 Structure: 0	Primary care delivered by GPs	Residents of care homes and community-dwelling people aged 65 to 104 who were registered for at least 90 days with their general practitioner. Excluded: patients with exceptions (including disease wide exceptions, contraindications for a specific intervention or unavailability of a service or refusal of a specific intervention). (n = 403,259 people in the community; n = 10 387 residents in care homes)
**Neumark, 2015** [49]	Sweden	Diabetes care quality according to national guidelines	Total: 16 Outcome:6 Process: 10 Structure: 0	Primary health care centers	Elderly people aged 80 and older, with diabetes living at home with home health care (compared with elderly without home health care and residents of nursing homes). Excluded: patients who were no longer residents of the municipality, or with an incorrect diagnosis of diabetes, or entered a palliative phase or died. (n = 277)
**Van der Ploeg, 2008** [46] Applied in: *Askari*, *2016* ^f^ *[44]*	Netherlands	General practice care quality for vulnerable elders (focus on 8 conditions that are associated with the development of frailty)	Total: 81^g^ Outcome: 0 Process: 81 Structure: 0	General practice care	Vulnerable elders, defined as community-dwelling individuals aged 65 and older who are at greater risk of death or functional decline over a 2-year period. (n = 950 [44])
*Other community care settings*					
**Vickrey, 2006** [34] *Applied in*: *Chodosh*, *2012* [25]	USA	Adherence to dementia guidelines recommendations (assessment, treatment, education and support, safety)	Total: 22 Outcome: 0 Process: 22 Structure: 0	Primary care clinics and community agencies	Patients with dementia aged 65 and older and their informal caregivers Excluded: patients without an informal caregiver aged 18 years or older. (n = 408 [34]; n = 238 [25])
**Kim, 2011** [28] *Applied in: Lemus, 2010 [30]* (AHRQ Prevention QIs)^h^ [51]	USA	Potentially preventable hospitalization for ambulatory care sensitive conditions (i.e. hospitalizations that may be preventable with high quality primary and preventive care)	Total: 8 Outcome: 12 Process: 0 Structure: 0	Outpatient care	Elders aged 65 years and older Excluded: hospitalizations of patients at psychiatric, rehabilitation, and long-term hospitals, and patients transferred from other institutions and/or discharged against medical advice, as defined by the AHRQ-PQI algorithm. (n = 555,538 [28]; n = 66,421 [30])
**Wenger, 2007** [36] *Applied in*: *Wenger*, *2011* [35]; *Reuben*, *2013* [32]; *Roth*, *2012* [33]; *Ganz*, *2010* [26]; *Jennings*, *2016* [27] (ACOVE-3 Indicators)	USA	Process-of-care quality of the medical care provided to vulnerable elders (26 conditions most important to vulnerable elders)	Total: 342 ^I^ Outcome: 0 Process: 342 Structure: 0	Community care	Vulnerable elders, defined as community-dwelling individuals aged 65 and older who are at greater risk of death or functional decline over a 2-year period. (n = 485 [32]; n = 231 [33]; n = 200 [26]; n = 797 [27])
**Kröger, 2007** [41]	Canada	Care and services quality for vulnerable older adults with cognitive impairment or dementia	Total: 72 ^j^ Outcome: 0 Process: 72 Structure: 0	Integrated community care	Vulnerable older adults with cognitive impairment/dementia and being treated in an integrated service system. The pilot study included 40 community-dwelling patients aged 75 and older with a diagnosis of cognitive impairment/dementia receiving home care services. (n = 40)

Abbreviations: QI, Quality Indicator; AHRQ, Agency for Healthcare Research & Quality

^a^ The interRai-HCQI set has been used worldwide in countries participating in the InterRai network

^b^ Two quality measures were excluded as these were not expressed with a numerator and denominator.

^c^ Two structural QIs used in this study were only applied in the nursing home setting, and therefore not included in this review

^d^ The following 8 European countries participated in this study: England, Estonia, Finland, France, Germany, the Netherlands, Spain, Sweden.

^e^ One QI used in this study to assess the subjective quality of home care was not expressed with a numerator and denominator, and therefore not included in this review.

^f^ Askari et al. (2016) used 9 (out of the 12 original) ACOVE fall-related QIs that were slightly adapted for the Dutch primary care setting by Van der Ploeg et al. (2008)

^g^ Van der Ploeg et al. (2008) described a shortened and adapted version of the ACOVE-3 QI set for Dutch primary care. In S2 Appendix, we only extracted the QIs which were newly added (n = 5) or changed significantly (n = 4) compared with the original set. The (highly) overlapping QIs are only listed in the original set (Wenger et al., 2007).

^h^ Although these QIs were measured with hospital inpatient data, they are aimed to provide insight into the quality of the ambulatory health care system, and seen by the developers as indirect measures of access to health care and quality of primary care in a community.

^i^ The total set included 392 QIs. Of these, 50 QIs focused on elders receiving hospital or nursing home care and were thus excluded for this study.

^j^ The 57 QIs from the ACOVE indicator set are not presented in the overview of the QIs (S2 Appendix), because the updated ACOVE set was extracted from Wenger et al. (2007).

Eleven articles used QIs from the Assessing Care Of Vulnerable Elders-3 set (ACOVE-3), a comprehensive set of indicators specifically developed to assess the medical care provided to vulnerable elders and covering a wide variety of conditions [36]. Four of these studies used an adapted version or part of the ACOVE indicators, in combination with other QIs [39, 41, 45, 46].

Of the 17 sets, five targeted persons with dementia or cognitive impairments [34, 40, 41, 45, 47], one assessed the care for older persons with diabetes [49], and one focused on older persons with chronic diseases (coronary heart disease, stroke, atrial fibrillation, and diabetes) [39]. The other sets were developed or applied in (frail) older samples, without focusing on a specific disease. The quality aspects that these QIs sets aimed to address varied, and included, for example, communication between physicians, potentially preventable hospitalization for ambulatory care sensitive conditions, home care service user experiences, and the quality of medication prescribing. The majority of the sets (10 out of 17) included one type of indicators. Only the set of Perry et al. (2010) [45] covered process, outcome and structure indicators (Table 1).

### Characteristics of the quality indicators

The Excel spreadsheet (S2 Appendix) shows the description of the 567 QIs, their numerator and denominator, the domain(s) they covered, the type (process, outcome or structure), and the specific community setting in which the QI was developed or applied. When applicable, the exclusion criterion, its performance standard, and the condition/ disease the indicator addresses are listed. Table 2 shows that 455 QIs (80%) referred to processes of care. A considerably smaller number of indicators measured the structure (n = 59, 10%) or outcome of care (n = 53, 9%). Most indicators (n = 355) assessed clinical issues, mainly with regard to follow-up, monitoring, examinations and treatment (Table 2), followed by indicators that evaluated the quality of care in the domain ‘Cognition or mental health’ (n = 67), ‘Structure of care’ (n = 59), and ‘Continuity and coordination of care’ (n = 36). About two third of the QIs focussed on a specific disease or were applied in a patient group with a particular disease (Table 2), mostly for people with dementia (n = 138), followed by several types of cancer (n = 65) and cardiovascular diseases (n = 61). Table 2 provides also insight in the QIs per domain and disease across the primary care, home care and other community care settings. The majority of QIs (68%) were developed or applied for community care (not further specified), or for a combination of community care providers. This was mainly due to the extensive number of QIs from the ACOVE indicator set represented within this category. Furthermore, respectively 17% and 14% of the QIs were developed or applied in home care and primary care settings.

**Table 2:** Number of quality indicators per domain and disease, presented by type of indicator, and by type of community care setting.

Domain	Type of indicator			Total*	Community care setting			Total*
	*Outcome*	*Process*	*Structure*		*Home care*^c^	*Primary care*^d^	*Other*^e^	
Clinical issues	27	328	0	355	18	53	284	355
Screening and prevention	0	21	0	21	1	6	14	21
Medication	3	97	0	100	1	16	83	100
Non-pharmacological treatment	0	90	0	90	0	2	88	90
Clinical events and targets	24	3	0	27	16	8	3	27
Follow-up, monitoring and examinations	0	134	0	134	0	22	112	134
Cognition/ Mental health	7	59	1	67	7	16	44	67
Structure of care	0	0	59	59	54	4	1	59
Continuity and coordination of care	0	35	1	36	3	9	24	36
Psycho-social aspects	3	23	1	27	3	5	19	27
Functional performance	4	22	0	26	5	1	20	26
End of life	0	18	0	18	0	0	18	18
Patient perceptions and interaction	0	15	0	15	6	2	7	15
Service utilization	12	2	0	14	3	1	10	14
**Disease/ condition**								
Not disease-specific	31	149	2	182	39	13	130	182
Dementia	7	74	57	138	60	24	54	138
Cancer^a^	0	65	0	65	0	0	65	65
Cardiovascular^b^	0	61	0	61	0	11	50	61
Diabetes Mellitus	15	29	0	44	0	25	19	44
Depression	0	25	0	25	0	6	19	25
COPD	0	12	0	12	0	1	11	12
Falls and mobility disorders	0	12	0	12	0	0	12	12
Hypothyroidism	0	1	0	1	0	1	0	1
Osteoarthritis	0	20	0	20	0	0	20	20
Sleep disorders	0	10	0	10	0	0	10	10
Pulmonary disease	0	4	0	4	0	0	4	4
Renal and liver diseases	0	2	0	2	0	0	2	2
***Total per QI type***	53 (9%)	455 (80%)	59 (10%)					
***Total per setting***					99 (17%)	81 (14%)	387 (68%)	

*Quality Indicators could be included in more than one (sub)domain or cover more than one condition.

^a^ included: Benign prostatic hyperplasia, breast cancer, colorectal cancer, lung cancer, cancer (not specified).

^b^ included: Congestive heart failure, stroke, myocardial infarction, atrial fibrillation, coronary artery disease, coronary heart disease, heart disease, heart failure, ischaemic heart disease, ischaemic stroke, myocardial infarction, stroke, stroke and atrial fibrillation.

^c^ [29, 38, 40, 42, 43, 47, 48]

^d^ [31, 37, 39, 45, 46, 49]

^e^ included: a combination of community care agencies and primary care clinics [34], outpatient care [28] and community care settings not further specified [36, 41]

### Methodological quality of the QI sets

Table 3A and 3B present the results of the methodological assessment of the 17 QI sets as assessed with the AIRE Instrument. Overall, the sets scored highest on the domain, ‘Additional evidence, formulation and usage’ (domain score of 51%), with 53% of the studies within the high quality level (i.e. a score of 50% or higher). The methodological level in terms of ‘Stakeholder involvement’ and ‘Scientific evidence’ was lower, with mean domain scores of respectively 28% and 39%, and with 41% and 18% of the studies meeting the high-quality threshold in these domains. In most studies, the target patient population of the indicators was clearly defined, and the numerator and denominator were described in detail (mean item scores of 3.5 and 3.3). Also, indicators were frequently based on recommendations from an evidence-based guideline (mean score 2.9). Furthermore, the QIs were, to some extent, piloted in practice, and efforts needed for data collection were quite well considered (mean scores 2.7). In contrast, indicators were rarely formally endorsed (mean score 1.4), hardly appraised the supporting evidence critically (mean score 1.6) and had demonstrated sufficient reliability to a limited extent (mean score 1.7).

**Table 3:** Methodological characteristics of the quality indicator sets assessed with the AIRE instrument ^a^.

**A**									
Item^b^ (1–4); domain (%) score^c^:	Venables [40]	Kogan [29]	Foebel [48]	Jones [38]	Chang [42]	Kajonius [43]	Beerens [47]	Fahey [37]	Lund [31]
The group developing the indicator includes individuals from relevant professional groups	1	1	3	1	1	1	1	1.5	4
Considering the purpose of the indicator, all relevant stakeholders have been involved at some stage of the development process	1	1	3.5	1	1	1	1	1.5	3
The indicator has been formally endorsed	1.5	1	3	2.5	1	2	1	1.5	1
**Domain 1: Stakeholder involvement**	**6**	**0**	**72**	**17**	**0**	**11**	**0**	**17**	**56**
Systematic methods were used to search for scientific evidence	1	2.5	1.5	1	1.5	1	2.5	1.5	2.5
The indicator is based on recommendations from an evidence-based guideline	3.5	2.5	2	1.5	2	2.5	3	1.5	3.5
The supporting evidence has been critically appraised	1	1	1	1	1	1	1.5	1	3
**Domain 2: Scientific evidence**	**28**	**33**	**17**	**6**	**17**	**17**	**44**	**11**	**67**
The numerator and denominator are described in detail	3	4	4	2	3.5	4	2.5	3.5	2
The target patient population of the indicator is defined clearly	2.5	3	3.5	3	4	3.5	4	3.5	4
A strategy for risk adjustment has been considered and described	1	1	4	1	2.5	1.5	1	3	2.5
The indicator measures what it is intended to measure (validity)	1	1.5	3.5	1.5	1	1	1.5	1	2.5
The indicator measures accurately and consistently (reliability)	1	1.5	3.5	2.5	1	1	1	1	1
The indicator has sufficient discriminative power	1.5	1.5	4	1	3.5	2.5	2.5	2.5	3
The indicator has been piloted in practice	3	3	3,5	3	3	2,5	3	3	2,5
The efforts needed for data collection have been considered	2.5	2.5	2	2.5	3.5	2.5	2	2.5	2.5
Specific instructions for presenting and interpreting the indicator results are provided	2	3.5	3	3	3	3	3	3	2.5
**Domain 3: Additional evidence, formulation and usage**	**31**	**46**	**81**	**39**	**59**	**46**	**43**	**52**	**50**
**B**									
Item^b^ (1–4); domain (%) score^c^:	Perry [45]	Shah [39]	Neumark [49]	Van der Ploeg [46]; Askari [44]	Vickrey [34]; Chodosh [25]	Kim [28]; Lemus [30]	Wenger [36]; Reuben [32]; Wenger [35]; Roth [33]; Ganz [26]; Jennings [27]	Kröger [41]	*Mean score all sets*
The group developing the indicator includes individuals from relevant professional groups	4	2.5	1	4	3	3	4	4	*2*.*2*
Considering the purpose of the indicator, all relevant stakeholders have been involved at some stage of the development process	4	2.5	1	3	3.5	2,5	3	3	*2*.*0*
The indicator has been formally endorsed	1	1.5	1.5	1	1	1	1	1	*1*.*4*
**Domain 1: Stakeholder involvement**	**67**	**39**	**6**	**56**	**50**	**39**	**56**	**56**	***28***
Systematic methods were used to search for scientific evidence	2.5	2.5	1.5	2.5	1.5	4	4	2	*2*.*1*
The indicator is based on recommendations from an evidence-based guideline	3.5	2.5	3.5	3	4	3.5	4	3.5	*2*.*9*
The supporting evidence has been critically appraised	1	1	1	1.5	1.5	4	3.5	1.5	*1*.*6*
**Domain 2: Scientific evidence**	**44**	**33**	**33**	**44**	**44**	**94**	**94**	**44**	***39***
The numerator and denominator are described in detail	3.5	4	2.5	3.5	2.5	3.5	4	3.5	*3*.*3*
The target patient population of the indicator is defined clearly	2.5	4	4	3	4	3.5	4	3	*3*.*5*
A strategy for risk adjustment has been considered and described	1	3	1	1	2.5	3.5	2.5	1	*2*.*0*
The indicator measures what it is intended to measure (validity)	3.5	3	1	3.5	1.5	3.5	4	4	*2*.*2*
The indicator measures accurately and consistently (reliability)	4	1	1	1	3	2	2.5	2.5	*1*.*7*
The indicator has sufficient discriminative power	3.5	3	2.5	1	3	2.5	2	1	*2*.*2*
The indicator has been piloted in practice	3,5	2,5	3	1,5	3	2,5	2,5	3	*2*,*7*
The efforts needed for data collection have been considered	2.5	3	3.5	1.5	2	4	2.5	2.5	*2*.*7*
Specific instructions for presenting and interpreting the indicator results are provided	1	3	2.5	1	3	3	3	1	*2*.*6*
**Domain 3: Additional evidence, formulation and usage**	**59**	**65**	**44**	**30**	**57**	**70**	**67**	**46**	***51***

Abbreviations: AIRE, Appraisal of Indicators through Research and Evaluation

^a^ Available at: http://zorginzicht.garansys.nl/kennisbank/PublishingImages/Paginas/AIRE-instrument/AIRE%20Instrument%202.0.pdf. The complete AIRE Instrument contains a fourth category ‘‘Purpose, Relevance and Organizational Context,” which was not used in this review [15].

^b^ 1 = “strongly disagree” (criterion was not met or no information was provided); 2–3 = “agree/ disagree” (not sure if the criterion was met); 4 = “strongly agree” (criterion was met).

^c^ The domain scores were calculated with the formula: (total score—minimum possible score) / (maximum score—minimum possible score) x 100%. A higher standardized score indicates a higher methodological level (range 0–100%).

The methodological quality of the QI sets varied. Two QI sets were considered to have a high methodological quality on all three quality domains [31, 36], and four reached this level on two of the domains [28, 34, 45, 48]. The interRai-Home Care QI set [48, 50] scored highest on the domains ‘Stakeholder involvement’ and ‘Additional evidence, formulation and usage’. The Agency for Healthcare Research and Quality (AHRQ) prevention QI set [28, 51] and ACOVE-3 indicators set [36] achieved the best score on the domain ‘Scientific evidence’.

## Discussion

Through a systematic review of the literature, we identified 17 QI sets that covered a substantial number of 567 QIs, developed or applied to assess the quality of care for older people in the community. The majority of these QIs assessed processes of care (80%), and measured clinical issues (63%). Most QIs focussed on a specific disease. Although we identified a high number of indicators, there was some overlap in the content of QIs. For example, QIs for diabetes care measured the same aspects to evaluate whether physicians monitored their patients according to national guidelines or standards [36, 37, 39, 49]. Furthermore, several studies used indicators that were based on the comprehensive set of QIs developed by the ACOVE group, but modified the indicators to enable application in another country or by another community care provider [45, 46].

In terms of methodological quality, overall, the target population of the indicators was clearly defined, numerators and denominators were described in sufficient detail and indicators were based on evidence- based recommendations. On the other hand, there is still room for improvement, particularly with regard to the extent to which supporting evidence was critically appraised during the development process, a sufficient demonstration of the QIs’ reliability, and formal endorsement of QI sets. Besides, taking account of the purpose of the indicators, not all relevant stakeholders were involved in the development process in many of the studies, and a strategy for risk adjustment was frequently not considered or described. Often, studies did not describe these aspects at all or did not provide enough detail to obtain a good score.

There was considerable variability in the methodological quality between indicator sets. Six of the 17 sets (35%) were found to have a high methodological quality on at least two of the three quality domains [28, 31, 34, 36, 45, 48]. The ACOVE and AHRQ indicator sets almost achieved the maximum score on the domain ‘Scientific evidence’ and described in detail which methods were used to search for scientific evidence and how the evidence was appraised and supported the selection of the indicators. The interRai-HC set provided the best evidence in terms of reliability, and discriminative power, and used a thorough risk adjustment method. Risk adjustment is particularly important for outcome measures, because patient outcomes are not just determined by quality of care but also by patient characteristics, such as age, and level of impairment. Without adjusting for the effects of patient characteristics that may vary across providers, this could lead to incorrect conclusions about the quality of care, because organizations or providers with the worst outcomes may also have the most severely impaired patients. Stakeholders, such as health care insurers and the Health Inspection who may judge the quality of care based on QIs and use this information to make future decisions should strongly take into account whether QI outcome scores were correctly adjusted for differences in case mix. Besides, risk-adjusted quality measures creates the opportunity for benchmarking within and between countries and identify best practices. The high number of process indicators with, generally, a clearly defined target patient population may have reduced the need for risk adjustment.

### Strengths and limitations

To our knowledge, this is the first review that provides an overview of the QIs that are available to assess the quality of community care for older people and assessed their methodological quality. We systematically searched the literature in five electronic reference databases and thoroughly reviewed and evaluated a vast number of articles. The selection of articles, data extraction and quality assessment was conducted by two reviewers independently, which increases the reliability of the results. We included different types of community care settings, such as general practice and home care services. In addition to sets that were specifically developed for older people, we also included existing QIs that were used in older samples with the explicit goal of measuring the quality of care in older people. Therefore, we can be confident that this review provides a comprehensive overview of the available indicators. This can potentially be used to assess the quality of care for older people in the community and supports stakeholders to navigate through the QIs and to select QIs for their specific situation and purposes. The supplementary Excel spreadsheet (S2 Appendix) enables readers to filter QIs in a particular community care setting, care domain or for a specific disease.

Although this systematic review makes a significant contribution to the quality of care literature, some limitations must be acknowledged. First, despite the wide scope and substantial number of identified QIs, some (sets of) indicators could have been missed. The searches in the international literature databases mainly identified scientific research papers. We attempted to track down relevant grey literature through manually checking the reference lists during the full-text screening and data extraction phase, soliciting colleagues who investigate the quality of community care for older people about missing relevant QI sets, and using Google’s internet search engine when links to webpages did not work anymore. Nevertheless, this could not avoid that QI sets that have not been published in an article or report, or were published in another language than we could understand were not found. On the other hand, it is not very likely that QI sets which are well validated and reliable have not published yet in peer-reviewed literature. In addition, following the third inclusion criterion, QIs which were expressed as a continuous measure, such as some summary measures, or satisfaction measures expressed with a scale score were excluded.

Second, this review mainly captured QIs which measured quality of care from the perspective of the care provider. Over the past years, there has been a growing interest to involve the patient’s perspective to inform health care quality improvement. For example, patients are asked about the impact of treatments and care on their health through the use of patient-reported outcome measures (PROMs). Besides, patient experience measures (PREMs) are used to assess patient satisfaction with a health care service [52]. A few of these were included in our review within the domain “Patient perceptions and interaction”. The absence of PROMs and limited number of PREMs that were selected could suggest that these measures have not yet been widely implemented in geriatric care and are more common in adult patient groups. This is in line with findings from a recent literature review which reported that the implementation of PROMs is most advanced in specific settings or disease-groups [53]. It could also be possible that papers described these measures in other words than included in our search terms and might have been missed, although we used a broad range of terms, such as for example ‘health care quality’, ‘treatment outcome’ and ‘care performance’ (S1 Appendix). As PROMs and PREMs are often measured with self-completed questionnaires, and expressed as a score, they might also have been excluded because one of our inclusion criterion required that the numerator and denominator of the QIs were defined or could be deduced from the description. Nevertheless, when collected systematically across providers, and measured in a valid and reliable way, PROMs can generate valuable data to improve quality and support patient-centered care.

Lastly, as mentioned, the methodological quality of the QI sets could have been underestimated to some extent. Following the instructions from the AIRE instrument, the lowest score was assigned on an item if no information was provided in the publication. Particularly the development process of the indicators and the evidence on these were based were not always described, or sufficient details were lacking, while the AIRE instrument puts relatively much emphasis on development aspects. Also, information about formal endorsement of QIs was barely available in the included articles. Research papers may put less emphasize on this type of information, which may have resulted in lower quality scores on these aspects. We have tried to resolve this by incorporating as much information as possible about the indicator sets when evaluating their quality. For example, we examined the relevant references and searched the internet. However, it was not always possible to find a report or website link from the reference lists, or to obtain enough detail about the development process from the references.

### Conclusion and implications

This systematic literature review shows that, over the last decades, a substantial number of QIs has been developed or applied to assess the quality of care for older people in the community. When monitoring the quality of care, it would be useful for policy makers, researchers, clinicians and other relevant stakeholders to first consider the QIs that are already available before developing new indicators. Given the variation in methodological quality and rather low scores on some aspects, a priority could be to further improve the existing indicators. For example, the supporting evidence can be appraised more critically, QIs can be further tested in daily practice, adapted for use in other countries and the efforts needed for data collection can be decreased where possible. Currently, process QIs, focusing on clinical aspects and specific diseases are overrepresented. While the tendency to measure care performance is shifting from process to (patient-reported) outcome measures, this review shows that valid outcome indicators for the quality of care for older people are still relatively limited. It would be desirable to find a better balance between measuring processes and outcomes. Both types of indicators have their particular strengths and weaknesses, depending on the purpose for which the indicator is used and by whom [11, 12]. For clinicians, process indicators could be more interesting as these are direct measures of quality and give straightforward information. It may thus be more clear what action needs to be taken to improve the quality of care. The interpretation of differences in outcome indicators can be more difficult, as alternative explanations should be considered before it can be concluded that the difference truly reflects variations in the quality of care [11]. However, for other stakeholders, such as health care insurers or patients, the cause of differences in quality of care may be less importance and outcomes are likely to be more interesting. In this review, we identified only one QI set included a mix of process, outcome and structure [45]. As processes, outcomes and the structure of care are related to each other, we would recommend to consider all types of QIs when measuring the quality of care In addition, the findings suggest that more attention can be paid to non-clinical domains. Particularly for frail older people, remaining functionally stable, and living at home as long as possible in good (psycho-social) health are just as important as the treatment of medical problems. Lastly, generic indicators, measuring aspects of care that are relevant to most older patients (e.g. preventing acute hospitalization, loneliness, pain), were underrepresented. The stakeholder groups should realize these gaps when developing or utilizing QIs to optimize the care for older people in the community.

## Supporting information

S1 Appendix Search strategy in Medline. (PDF)

S2 Appendix List of quality indicators. (XLSX)

S3 Appendix PRISMA checklist for systematic reviews. (PDF)
