# Supplementary material for: Quality indicators for community care for older people: A systematic review
Source: PLoS One. 2018 Jan 9;13(1):e0190298. doi: 10.1371/journal.pone.0190298 (PMC5760020; doi:10.1371/journal.pone.0190298)
Supplement: S1 Appendix — (PDF) [file pone.0190298.s001.pdf]

Appendix 1. Search strategy in Medline

Method: #1 AND #2 AND #3

#1 Quality indicators

"Quality Indicators, Health Care"[Mesh] OR "Quality Indicators, Health Care"[tiab] OR “quality measure”[tiab] OR “quality criterium”[tiab] OR “quality assesment”[tiab] OR “Healthcare quality indicator”[tiab] OR “Healthcare quality indicators”[tiab] OR “quality indicators”[tiab] OR “quality indicator”[tiab] OR “health care quality”[tiab] OR “process assessment”[tiab] OR “treatment outcome”[tiab] OR “quality of nursing care”[tiab] OR “care performance”[tiab] OR “care outcome”[tiab]

#2 Home care

"Home Care Services"[Mesh] OR “community care”[tiab] OR “primary care”[tiab] OR “Home care services”[tiab] OR “Home care service”[tiab] OR “Domiciliary care”[tiab] OR “Formal home care”[tiab] OR “district nursing”[tiab] OR “home care”[tiab] OR “domestic health care”[tiab] OR “domiciliary care”[tiab] OR "home care agencies"[MeSH Terms] OR “home care agencies”[tiab] OR "Home Care Services/organization and administration"[Mesh] OR "Home Health Aides/organization and administration"[Mesh] OR “long term care”[tiab]

#3 Elderly

"aged"[Mesh] OR "aged, 80 and over"[Mesh] OR "aged"[tiab] OR "aged, 80 and over"[tiab] OR “frail elderly”[tiab] OR “oldest old”[tiab] OR “older people”[tiab] OR “senior”[tiab] OR elder*[tiab]
